# Evaluation of Fucosylated Haptoglobin and Mac-2 Binding Protein as Serum Biomarkers to Estimate Liver Fibrosis in Patients with Chronic Hepatitis C

**DOI:** 10.1371/journal.pone.0151828

**Published:** 2016-03-22

**Authors:** Seiichi Tawara, Tomohide Tatsumi, Sadaharu Iio, Ichizou Kobayashi, Minoru Shigekawa, Hayato Hikita, Ryotaro Sakamori, Naoki Hiramatsu, Eiji Miyoshi, Tetsuo Takehara

**Affiliations:** 1 Department of Gastroenterology and Hepatology, Osaka University Graduate School of Medicine, Suita, Osaka, Japan; 2 Department of Gastroenterological Medicine, Hyogo Prefectural Nishinomiya Hospital, Nishinomiya, Hyogo, Japan; 3 Department of Gastroenterological Medicine, Higashiosaka City General Hospital, Higashiosaka, Osaka, Japan; 4 Department of Molecular Biochemistry and Clinical Investigation, Osaka University Graduate School of Medicine, Suita, Osaka, Japan; Saint Louis University, UNITED STATES

## Abstract

Fucosylated haptoglobin (Fuc-Hpt) and Mac-2 binding protein (Mac-2 bp) are identified as cancer biomarkers, based on the results from a glyco-proteomic analysis. Recently, we reported that these glyco-biomarkers were associated with liver fibrosis and/or ballooning hepatocytes in patients with nonalcoholic fatty liver disease (NAFLD). We evaluated the ability of these glycoproteins to estimate liver fibrosis in 317 patients with chronic hepatitis C. We measured the serum Fuc-Hpt and Mac-2 bp levels using a lectin-antibody ELISA and ELISA, respectively. The serum levels of both Fuc-Hpt and Mac-2 bp increased with the progression of liver fibrosis. The multivariate analysis revealed that Mac-2 bp was an independent factor associated with moderate liver fibrosis (F ≥ 2). In contrast, Fuc-Hpt was an independent factor associated with advanced liver fibrosis (F ≥ 3). In terms of evaluating liver fibrosis, the serum levels of these glycomarkers were correlated with well-known liver fibrosis indexes, such as the aspartate aminotransferase to platelet ratio index (APRI) and Fibrosis-4 (FIB4) index. An assay that combined the APRI or FIB4 index and the Fuc-Hpt or Mac-2 bp levels increased the AUC value for diagnosing hepatic fibrosis. Interestingly, the cumulative incidence of hepatocellular carcinoma (HCC) was significantly higher in the patients with elevated serum levels of Fuc-Hpt and Mac-2 bp. In conclusion, both Fuc-Hpt and Mac-2 bp could be useful glyco-biomarkers of liver fibrosis and predictors of HCC in patients with chronic hepatitis C.

## Introduction

Hepatitis C virus (HCV) infection is the largest cause of liver cirrhosis and hepatocellular carcinoma (HCC) in the world [1]. HCV infection gradually progresses from liver fibrosis to liver cirrhosis. It is known that the stage of liver fibrosis is one of the risk factors for the development of HCC. The stage of liver fibrosis could be a predictive factor for the patients’ response to interferon therapy [2–4]. Thus, the evaluation of the clinical stage of liver fibrosis is very important. Liver biopsy has been the gold standard for evaluating liver fibrosis. However, liver biopsy is invasive for patients and carries a small risk of life-threatening complications, such as bleeding. It is difficult to perform multiple liver biopsies to follow the degree of liver fibrosis. Thus, a non-invasive method for the assessment of liver fibrosis is required. Several markers, such as the platelet counts and hyaluronic acid, type IV collagen 7S and type III procollagen-N-peptide (P-III-P) levels, have been reported to be associated with liver fibrosis [5–7]. Recently, several combinations of biochemical markers, such as the aspartate aminotransferase to platelet ratio index to platelet ratio index (APRI), Fibrosis-4 (FIB4) index and FibroTest, have also been identified as providing a better evaluation of liver fibrosis [8–10]. It was reported that it was possible to define a group at high risk of developing HCC by intermittently measuring the FIB-4 index [11]. If a new type of biomarker is identified for liver fibrosis, the accuracy of diagnosis could be increased by the combined use of these known indexes.

A glycoprotein that contains a disease-related carbohydrate chain attracts attention for the development of the graphic equalizer proteomics technology. Fucosylation is one of the most important glycosylation events involved in cancer and inflammation [12]. Fuc-Hpt was observed in the fucosylated glycoproteins that were identified in the sera of patients with pancreatic cancer [13], and Mac-2 bp was identified as a hyperfucosylated protein in the conditioned medium of cancer cell lines. Although the changes in the biological functions of the haptoglobin and Mac-2 bp with fucosylation remain unknown, the serum levels of these glycoproteins have potential as cancer/inflammation-associated biomarkers. We recently demonstrated that the serum levels of Fuc-Hpt and Mac-2 bp could predict the presence of ballooning hepatocytes in patients with non-alcoholic fatty liver disease (NAFLD) [14, 15], and the serum Mac-2 bp levels are also associated with liver fibrosis as an independent factor from a multivariate analysis [15]. Because HCV infection is the major cause of liver cirrhosis, the accurate evaluation of liver fibrosis is important. However, the efficacies of these glycoproteins are unknown. In the present study, we examined the potential usefulness of Fuc-Hpt and Mac-2 bp in evaluating liver fibrosis in patients with chronic hepatitis C who underwent liver biopsy. We demonstrated that the serum levels of both Fuc-Hpt and Mac-2 bp were associated with liver fibrosis in patients with chronic hepatitis C, and the combined use of these biomarkers with known fibrosis markers exhibited clinical significance in evaluating liver fibrosis in the chronic hepatitis C patients. Furthermore, these markers might be able to predict the development of HCC.

## Materials and Methods

### Patients

Three hundred seventeen HCV-infected patients were enrolled in this study. All patients were infected with HCV and had a liver biopsy between January 2005 and October 2013 at Higashiosaka City General Hospital. The patients who were co-infected with hepatitis B virus (HBV) were excluded from this study. Blood samples were taken from all patients on the day of liver biopsy. This study was conducted in accordance with the Declaration of Helsinki, as amended in 2002. This clinical study was approved by the Institutional Review Boards (IRBs) of both Osaka University Hospital and Higashiosaka City General Hospital (No. 13214, accepted September 19, 2013).

All serum samples were collected at Higashiosaka City General Hospital. Some samples were collected under written informed consent, but some were under verbal informed consent. The collection of serum samples were approved by IRB of Higashiosaka City General Hospital. But in early days after starting collecting samples, verbal consent was provided for the serum sample preservation. The physician wrote the acceptance of verbal informed consent on the medical record in early days. After approving modified the clinical experimental documents, all samples were collected under written consent. Before analyzing samples, both IRBs of Osaka University Hospital and Higashiosaka City General Hospital were approved this consent procedure.

This study is a retrospective study and old serum samples were very precious. We consulted the use of samples under verbal informed consent to IRB at Osaka University Hospital. The IRB required that all preserved serum samples and all clinical information were handled in anonymity and all clinical information must be modified not to be able to identify each participant. All the information became anonymity at the time of submission to Osaka University from Higashiosaka City General Hospital. The clinical information was given new ID at Osaka University. We publicized the information of this study at Home Page and clearly showed the procedure for declining the use of samples if the participants wanted. Both IRBs of Osaka University Hospital and Higashiosaka City General Hospital required us these before starting this study and approved this study.

### Histological evaluation

Liver biopsies were taken by fine needle aspiration (16G sonopsy) guided by ultrasonography at Higashiosaka City General Hospital. The liver tissue specimens were fixed in 10% formalin and embedded in paraffin according to the standard procedure at the institution. The slices were stained with hematoxylin and eosin. They were evaluated for the stage of fibrosis and the grade of activity according to the criteria of the New Inuyma classification by pathologists at Department of Pathology, Higashiosaka City General Hospital. The liver fibrosis stage was classified as follows: F0 (no fibrosis), F1 (portal expansion), F2 (bridging fibrosis), F3 (bridging fibrosis with lobular distortion) and F4 (liver cirrhosis) [16]. In this study, we denoted the liver fibrosis as follows for convenience: F ≥ 2 (moderate fibrosis) and F ≥ 3 (advanced fibrosis).

### Clinical and biochemical data

The relevant clinical data included the patient’s age, gender, and alcohol consumption. We defined alcohol drinking as more than 20 g per day. The biochemical data were recorded at Higashiosaka City General Hospital and included the HCV RNA, HBs-antigen, anti-nuclear antibody, hemoglobin levels, platelet count, prothrombin time (PT), and total bilirubin (T-bil), aspartate aminotransferase (AST), alanine aminotransferase (ALT), serum total protein, serum albumin (Alb), hyaluronic acid and alpha fetoprotein (AFP) levels. We collected the clinical information after liver biopsy, including the history and viral responses to interferon treatment, follow-up period, development of HCC and prognosis.

### ELISA for Fuc-Hpt

The serum Fuc-Hpt levels were determined using a lectin-antibody ELISA kit developed by the Department of Molecular Biochemistry and Clinical Investigation, Osaka University Graduate School of Medicine. The Fab fragment of anti-human haptoglobin IgG (Dako, Carpinteria, Calif) was coated on the bottom of a 96-well ELISA plate. This IgG contains a fucosylated oligosaccharide in its Fc portion [17]. The coated plates were blocked with phosphate-buffered saline (PBS) containing 3% bovine serum albumin for one hour, followed by washing with PBS containing 0.1% Tween 20 (PBS-T). A 50 μL aliquot of sera was added to each well and incubated for one hour at room temperature. The plate was washed three times with PBS-T using Immuno Wash (Bio-Rad Model 1517, Tokyo, Japan). To detect Fuc-Hpt, a 1/1000 dilution of biotinylated Aleuria aurantia lectin (AAL) was added to each well, followed by incubation for one hour at room temperature. After the plates were washed three times with PBS-T, peroxidase-conjugated avidin was added to each well and incubated for one hour at room temperature. After four washes with PBS-T, tetramethylbenzidine was added to each well and incubated for 15 minutes to develop the reaction. To stop the development, 1 N sulfuric acid was added to each well. A standard curve for Fuc-Hpt was obtained as described [17] using conditioned medium from the PK8 pancreatic cancer cell line transfected with an expression vector for haptoglobin, which was purchased from Takara Bio Inc. (Shiga, Japan). The measurement values are reported as relative units (U/mL).

### ELISA for Mac-2 bp

We measured the serum Mac-2bp levels using an ELISA kit (Immuno-Biological Laboratory, Gunma, Japan, code #27362) [15]. The 96-well ELISA plates were coated with 100 μL of 100 mM carbonate buffer (pH 9.5) containing 0.5 μg of the capture antibody, from which the Fc region was eliminated by pepsin digestion overnight at 4 degrees centigrade. The plates were then washed with PBS containing 0.1% Tween 20 (PBS-T), and blocked with 200 μL of 1% (w/v) BSA in PBS containing 0.05% NaN_3_ overnight at 4 degrees centigrade. After washing twice with PBS-T, 100 μL of the test samples and recombinant human Mac-2 bp that had been gradually diluted with 1% BSA in PBS-T, which was used as a standard, were applied to each well of the coated microtiter plate in duplicate, and incubated for one hour at 4 degrees centigrade. After four washes with PBS-T, 100 μL of the HRP-conjugated detection antibody were applied to each well and incubated for 30 minutes at 4 degrees centigrade. Then, each well was washed five times with PBS-T, and 100 μL of the tetramethylbenzidine solution was added to the wells as a substrate and incubated for 30 minutes in the dark at room temperature. The reaction was terminated by adding 100 μL of 1 M H_2_SO_4_ to each well. The absorbance of the solution was determined at 450 nm using a microplate reader (E-Max; Molecular Devices, Sunnyvale, CA). A standard curve for Mac-2 bp was obtained as described [15] using a recombinant Mac-2 bp protein that had been purified from the conditioned medium of HEK293 cells transfected with Mac-2 bp expression vectors.

### Statistical analysis

The statistical analysis was conducted using JMP pro 10.0 software (SAS Institute Inc., Cary, NC). Kruskal-Wallis tests and Wilcoxon tests were used to assess whether there were any significant differences in the clinical or serological characteristics between groups. The diagnostic performances were assessed by analyzing the receiver operating characteristic (ROC) curves. The probabilities of a true positive (sensitivity) and a true negative (specificity) assessment were determined for the selected cutoff values and the area under the receiver operating characteristic curve (AUROC) was calculated for each index. The Youden index was used to identify the optimal cutoff points. Multivariate logistic regression analyses were conducted to identify the parameters that significantly contribute to the estimation of hepatic fibrosis. The diagnostic effectiveness of combinations of fibrosis markers were assessed using the logistic regression analysis and likelihood ratio test. The Kaplan-Meier method was used to assess the cumulative incidence of HCC, and the groups were compared using the log-rank test. The differences were considered statistically significant at P < 0.05.

## Results

### Patients’ characteristics

The characteristics of the patients at baseline were summarized in Table 1. The histological examination of the liver revealed that fibrosis grade F0 was present in 7 patients, F1 was present in 149 patients, F2 was present in 87 patients, F3 was present in 58 patients, and F4 was present in 16 patients. The median serum levels of Fuc-Hpt and Mac-2 bp were 559 U/ml and 1847 ng/ml, respectively. The patients’ clinical courses after liver biopsy were summarized in Table 2. The median follow-up period after liver biopsy is 2.4 years. HCC was detected in 19 patients during the follow-up period. Eleven patients died. The cause of death was HCC in 5 patients, liver failure in 3 patients and illness other than liver in 3 patients.

**Table 1:** Demographic, clinical, serological and virological characteristics of the 317 patients infected with HCV.

Factor	Number or Median [Interquartile range]
Age, years	57 [45–64]
Gender, male/female	167/150
Liver histology,	
F0/1/2/3/4	7/149/87/58/16
A0/1/2/3	5/178/133/1
HCV RNA, <4.9/≥5.0 LogIU/mL	34/283
Alcohol, none/drinking/unknown	99/73/145
HBsAg, +/-	0/317
Anti-nuclear antibody, <40/40≤/unknown	246/35/36
Hemoglobin, g/dL	14.2 [13.3–15.4]
Platelet count, ×10^4^/μL	15.7 [12.1–20.5]
Prothrombin time, %	92 [83–100]
Total bilirubin, mg/dL	0.7 [0.5–0.9]
Aspartate aminotransferase, U/L	52 [35–78]
Alanine aminotransferase, U/L	64 [41–101]
Serum total protein, g/dL	7.5 [7.2–7.9]
Serum albumin, g/dL	4.1 [3.9–4.3]
Blood urea nitrogen, mg/dL	13.8 [11.5–16.2]
Creatinine, mg/dL	0.67 [0.57–0.77]
Hyaluronic acid, ng/mL (missing value, n = 74)	65.7 [26.3–155.8]
Alpha fetoprotein, ng/mL	5.6 [3.3–11]
Fucosylated haptoglobin, U/mL	559 [304–1135]
Mac-2 binding protein, ng/mL	1847 [1022–3021]

**Table 2:** Clinical course of the 317 patients after liver biposy.

Factor	Number or Median [Interquartile range]
Follow-up period after liver biopsy, years	2.4 [1.0–4.9]
Interferon therapy, none/completed/cessation	13/272/32
Viral response, SVR/non-SVR/unknown	167/89/16
HCC incidence after liver biopsy, yes/no	19/298
Prognosis, death/living	11/306

SVR: sustained virological response.

### The serum levels of both Fuc-Hpt and Mac-2 bp were associated with liver fibrosis in patients with chronic hepatitis C

**Fig 1:**
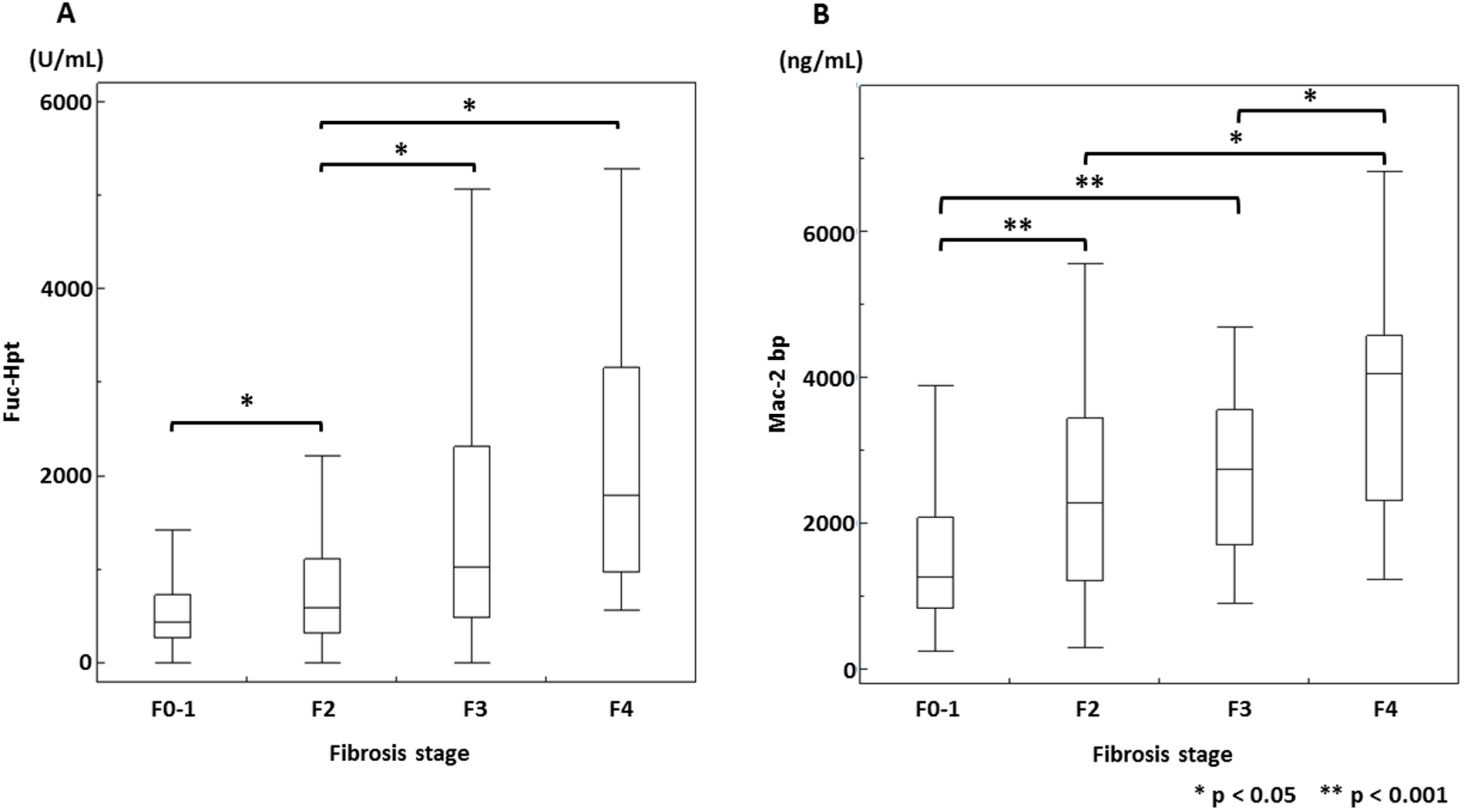
Box-and-whisker plot of the serum Fuc-Hpt (A) and Mac-2 bp (B) levels for each liver fibrosis stage. The top and bottom of each box represent the first and third quartiles, respectively, with the height of the box representing the interquartile range, covering 50% of the values. The line across each box represents the median. The error bars show the minimum and maximum values. The statistical analysis was performed with the Wilcoxon rank sum test. * P < 0.05. ** P < 0.001.

We compared the serum levels of Fuc-Hpt and Mac-2 bp with the liver fibrosis stages in patients with chronic hepatitis C (Fig 1). The median of serum Fuc-Hpt levels in patients with fibrosis grades F0–1, F2, F3, and F4 were 433, 592, 1022, and 1790 U/ml, respectively. The median of serum Mac-2 bp levels in patients with fibrosis grades F0–1, F2, F3, and F4 were 1261, 2276, 2740, and 4044 ng/ml, respectively. These results demonstrated that the serum levels of both Fuc-Hpt and Mac-2 bp increased with the progression of chronic liver diseases. The correlation coefficient between Fuc-Hpt and Mac-2 bp was 0.4307 (95% CI, 0.3365–0.5164), suggesting that there was a weak correlation between the serum Fuc-Hpt levels and those of Mac-2 bp.

### The usefulness of Fuc-Hpt, Mac-2 bp and conventional liver fibrosis markers for evaluating liver fibrosis

**Fig 2:**
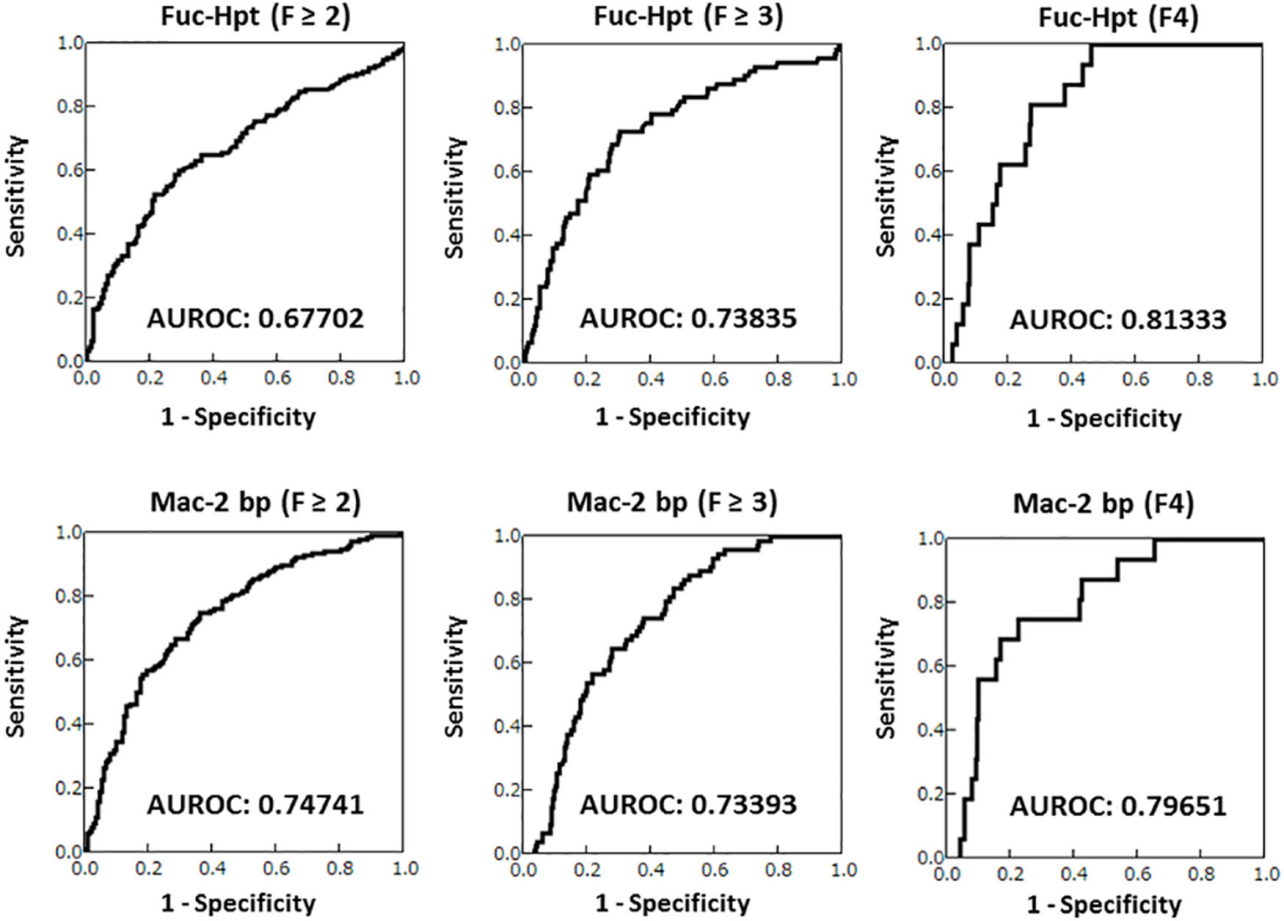
The diagnostic abilities of the serum Fuc-Hpt and Mac-2 bp levels for assessing the stage of liver fibrosis. The ROC curves and AUROC values of serum Fuc-Hpt and Mac-2 bp in F ≥ 2, F ≥ 3 and F4 stage liver fibrosis were shown in the figure.

We analyzed the following known liver fibrosis markers: platelet count, hyaluronic acid levels, APRI, and FIB4 index. The APRI was calculated using the following formula: (AST [U/L] / Upper limit of normal) x 100 / (platelet count [10^9^/L]) [18]. The FIB4 index was calculated as follows: (age [years] x AST [U/L]) / (platelet count [10^9^ /L] x (ALT [U/L])^1/2^) [9]. We compared the ability of these four fibrotic markers with that of the Fuc-Hpt and Mac-2 bp levels to evaluate moderate fibrosis (F ≥ 2), advanced fibrosis (F ≥ 3) and cirrhosis (F4). The ROC curves of the Fuc-Hpt and Mac-2 bp levels for the diagnosis of the F ≥ 2, F ≥ 3 or F4 fibrosis stages were shown in Fig 2. The area under the curve (AUROC) of Fuc-Hpt in F ≥ 2, F ≥ 3 and F4 were 0.67702, 0.73835 and 0.81333, respectively. The AUROC of Mac-2 bp in F ≥ 2, F ≥ 3 and F4 were 0.74741, 0.73393 and 0.79651.

The AUROC of the fibrosis markers that we could analyze in our study were summarized in Table 3. Because there were 74 missing values for hyaluronic acid, the hyaluronic acid analysis was performed in 245 cases as a reference. These known liver fibrosis markers are useful for evaluating liver fibrosis, as previously reported [5, 9, 18]. Most of the AUROCs of Fuc-Hpt and Mac-2 bp were more than 0.7 in all fibrotic stages, suggesting that both Fuc-Hpt and Mac-2 bp could be used for evaluating liver fibrosis.

**Table 3:** The diagnostic abilities of each liver fibrosis marker for assessing the stage of liver fibrosis.

	Fibrosis stage	Optimal cutoff	AUROC	Sensitivity (%)	Specificity (%)	PPV (%)	NPV (%)
**Fuc-Hpt, U/mL**	F ≥ 2	654	0.67702	59.0	72.4	68.8	63.1
	F ≥ 3	730	0.73835	73.0	70.0	42.5	89.5
	F4	966	0.81333	81.3	73.1	13.8	98.7
**Mac-2 bp, ng/mL**	F ≥ 2	1595	0.74741	75.2	64.1	68.4	71.4
	F ≥ 3	2352	0.73393	64.9	72.4	41.7	87.1
	F4	3029	0.79651	75.0	77.7	15.2	98.3
**Platelet count, ×10**^**4**^**/μL**	F ≥ 2	15.0	0.72135	59.0	74.4	70.4	63.7
	F ≥ 3	12.8	0.78893	66.2	79.4	49.5	88.5
	F4	9.4	0.85237	62.5	92.0	29.4	97.9
**Hyaluronic acid** *, ng/mL	F ≥ 2	75.6	0.77091	65.3	77.3	75.0	68.1
	F ≥ 3	85.0	0.80925	78.6	69.5	42.7	91.5
	F4	155.8	0.84591	78.6	78.2	18.0	98.4
**APRI**	F ≥ 2	0.65	0.76411	82.0	60.3	68.0	76.4
	F ≥ 3	0.86	0.78192	85.1	62.5	40.9	93.3
	F4	1.11	0.82828	87.5	66.4	12.2	99.0
**FIB4 index**	F ≥ 2	2.71	0.76867	63.4	79.5	76.1	67.8
	F ≥ 3	3.31	0.82710	70.3	82.3	54.7	90.1
	F4	5.83	0.87645	62.5	94.0	32.3	97.9

*The hyaluronic acid analysis was performed in 243 cases as a reference because 74 values were missing values. PPV: Positive predictive value. NPV: Negative predictive value.

### Identification of the factor associated with the liver fibrosis stage

We also identified the factors associated with the progression of liver fibrosis in patients with chronic hepatitis C. We excluded hyaluronic acid from this analysis because the values were missing for some patients. Because the FIB4 index strongly correlated with liver fibrosis in previous reports, we adopted this index, which is constructed of the AST, ALT, age and platelet count, and excluded these four markers. Because the AST and platelet count are components of both the APRI and FIB4 index, we also excluded the APRI from this analysis. In the multivariate analysis, the factors in F ≥ 2 were the Mac-2 bp levels, FIB4 index and AFP (Table 4). The factors in F ≥ 3 were the Fuc-Hpt levels, FIB4 index and AFP (Table 5). The factors in F4 were the Fuc-Hpt levels, FIB4 index and PT (Table 6). These results suggested that Mac-2 bp might be associated with early liver fibrosis stages. In contrast, Fuc-Hpt might be associated with advanced liver fibrosis stages.

**Table 4:** Factors associated with moderate liver fibrosis (F ≥ 2).

Factor	Category	Fibrosis stage	Univariate analysis			Multivariate analysis		
			OR	95% CI	*P* value	OR	95% CI	*P* value
Fuc-Hpt, U/mL	≤ 559 (median), > 559	F0-1 vs. F2-4	3.26	2.06–5.16	< 0.05	1.28	0.71–2.28	0.41
Mac-2 bp, ng/mL	≤ 1847 (median), > 1847	F0-1 vs. F2-4	4.44	2.77–7.12	< 0.05	2.23	1.27–3.92	< 0.05
Liver histology, activity	A0-1, A2-3	F0-1 vs. F2-4	2.08	1.32–3.27	< 0.05	1.38	0.80–2.36	0.25
FIB4 index	< 2.67, ≥ 2.67	F0-1 vs. F2-4	6.14	3.74–10.1	< 0.05	3.19	1.81–5.68	< 0.05
PT, %	≥ 80, < 80	F0-1 vs. F2-4	2.89	1.54–5.42	< 0.05	1.48	0.68–3.27	0.32
Alb, g/dL	≥ 4.0, < 4.0	F0-1 vs. F2-4	2.43	1.52–3.91	< 0.05	0.82	0.50–1.71	0.82
AFP, ng/mL	< 10, ≥ 10	F0-1 vs. F2-4	8.18	4.35–15.4	< 0.05	3.13	1.52–6.67	< 0.05

OR: odds ratio.

**Table 5:** Factors associated with advanced liver fibrosis (F ≥ 3).

Factor	Category	Fibrosis stage	Univariate analysis			Multivariate analysis		
			OR	95% CI	*P* value	OR	95% CI	*P* value
Fuc-Hpt, U/mL	≤ 559 (median), > 559	F0-2 vs. F3-4	5.01	2.53–9.22	< 0.05	2.19	1.02–4.77	< 0.05
Mac-2 bp, ng/mL	≤ 1847 (median), > 1847	F0-2 vs. F3-4	3.87	2.17–6.91	< 0.05	1.13	0.53–2.41	0.74
Liver histology, activity	A0-1, A2-3	F0-2 vs. F3-4	2.32	1.36–3.94	< 0.05	1.33	0.71–2.41	0.38
FIB4 index	< 2.67, ≥ 2.67	F0-2 vs. F3-4	8.17	4.36–15.3	< 0.05	3.47	1.70–7.28	< 0.05
PT, %	≥ 80, < 80	F0-2 vs. F3-4	2.87	1.55–5.30	< 0.05	1.06	0.49–2.23	0.88
Alb, g/dL	≥ 4.0, < 4.0	F0-2 vs. F3-4	4.29	2.48–7.42	< 0.05	1.75	0.89–3.43	0.11
AFP, ng/mL	< 10, ≥ 10	F0-2 vs. F3-4	7.44	4.18–13.2	< 0.05	2.50	1.21–5.17	< 0.05

**Table 6:** Factors associated with liver cirrhosis (F4).

Factor	Category	Fibrosis stage	Univariate analysis			Multivariate analysis		
			OR	95% CI	*P* value	OR	95% CI	*P* value
Fuc-Hpt, U/mL	≤ 559 (median), > 559	F0-3 vs. F4	–	–	< 0.05	2.43 <	2.43 -	< 0.05
Mac-2 bp, ng/mL	≤ 1847 (median), > 1847	F0-3 vs. F4	7.53	1.68–33.7	< 0.05	0.96	0.17–7.56	0.97
FIB4 index	< 2.67, ≥ 2.67	F0-3 vs. F4	21.7	2.83–166	< 0.05	4.24	0.58–89.5	0.17
T-bil, mg/dL	≤ 1, > 1	F0-3 vs. F4	8.29	2.96–23.8	< 0.05	2.89	0.80–10.4	0.10
PT, %	≥ 80, < 80	F0-3 vs. F4	12.5	4.15–37.7	< 0.05	5.08	1.34–21.7	< 0.05
Alb, g/dL	≥ 4.0, < 4.0	F0-3 vs. F4	4.27	1.44–12.6	< 0.05	0.44	0.10–1.92	0.27
AFP, ng/mL	< 10, ≥ 10	F0-3 vs. F4	22.5	4.99–101	< 0.05	4.19	0.82–33.9	0.09

### The diagnostic ability of the combination of the Fuc-Hpt and Mac-2 bp levels with the FIB4 index

It is possible that the combination of useful markers might be effective in establishing better diagnostic liver fibrosis markers. We examined the correlation coefficients between these markers (Table 7). The correlation coefficients between the Fuc-Hpt or Mac-2 bp levels and the FIB4 index were less than 0.4, suggesting that the correlations between the Fuc-Hpt or Mac-2 bp levels and the FIB4 index were weak. As mentioned above, the factors associated with liver fibrosis in F ≥ 2 were the Mac-2 bp levels and the FIB4 index (Table 4), and the Fuc-Hpt levels and FIB4 index in F ≥ 3 (Table 5). Therefore, we analyzed the Logit (p) of these combinations using a logistic regression analysis. The combinations of the FIB4 index with the Mac-2 bp levels in F ≥ 2 and with the Fuc-Hpt levels in F ≥ 3 were better than each single marker (Table 8). The effectiveness of these combinations was significant using the likelihood ratio test. These results demonstrated that the combinations using the Fuc-Hpt and Mac-2 bp levels may be new biomarkers for evaluating liver fibrosis.

**Table 7:** The correlation coefficients between each of the liver fibrosis markers.

	Correlation coefficients				
vs.	Mac-2 bp	Platelet count	Hyaluronic acid	APRI	FIB4 index
Fuc-Hpt	0.4312	- 0.2469	0.4701	0.2962	0.3153
Mac-2 bp		- 0.2627	0.2524	0.2715	0.3103
Platelet count			- 0.4033	- 0.5821	- 0.7318
Hyaluronic acid				0.3711	0.5196
APRI					0.7777

**Table 8:** The diagnostic abilities of combinations with these glycoproteins.

	Fibrosis stage	AUROC	Logit (p)	P value; by the Likelihood ratio test
FIB4	F ≥ 2	0.76867	- 0.7205 + 0.6598×(FIB4)	
FIB4 + Mac-2 bp	F ≥ 2	0.80005	- 1.198 + 0.5413×(FIB4) + 0.0003561×(Mac-2 bp)	FIB4; p < 0.0001, Mac-2 bp; p = 0.0003
FIB4	F ≥ 3	0.82710	- 2.244 + 0.6166×(FIB4)	
FIB4 + Fuc-Hpt	F ≥ 3	0.84079	- 2.327 + 0.5610×(FIB4) + 0.0001962×(Fuc-Hpt)	FIB4; p < 0.0001, Fuc-Hpt; p = 0.0305

### The cumulative hepatocellular carcinoma incidence rate

**Fig 3:**
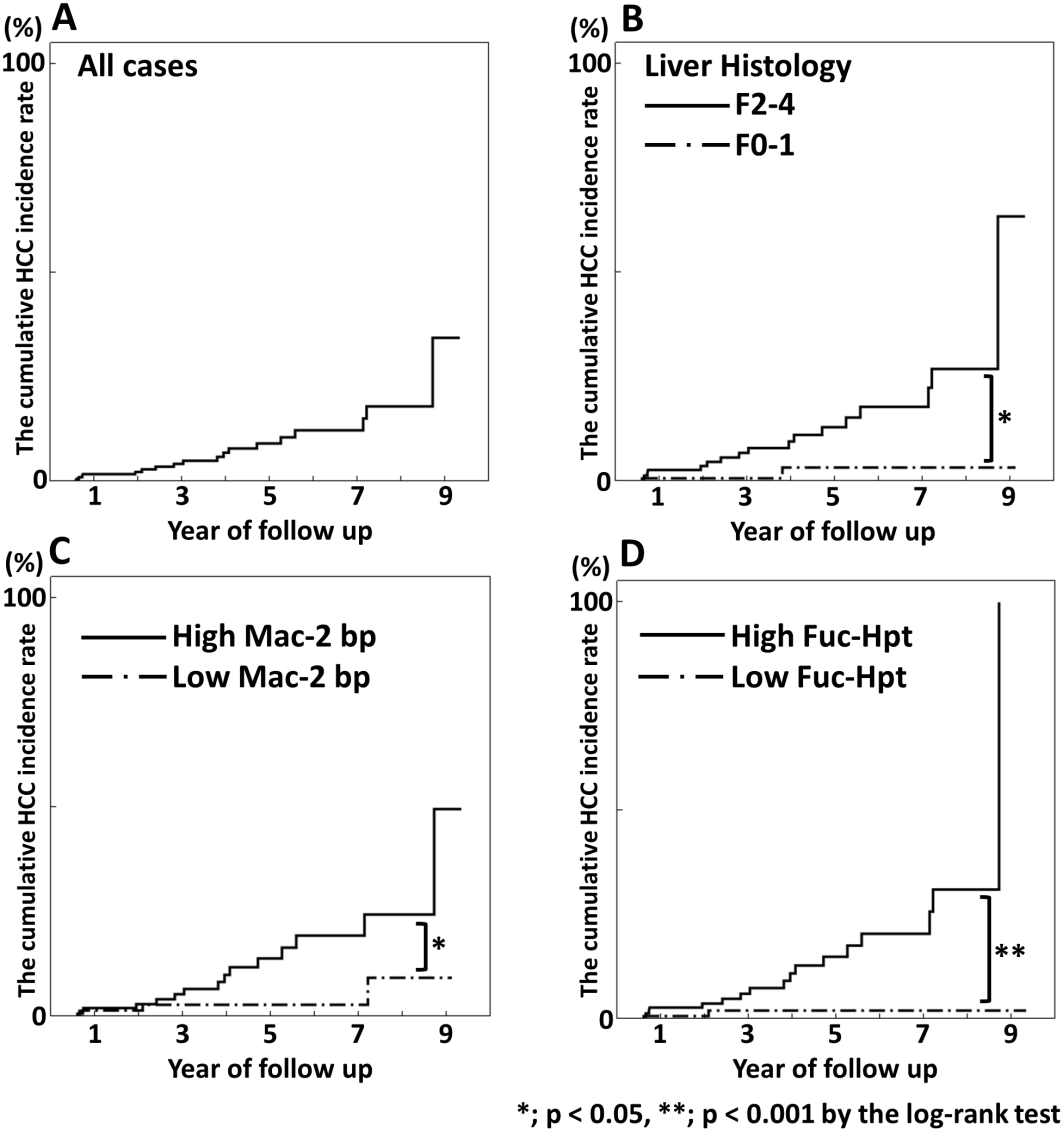
The cumulative hepatocellular carcinoma incidence rate. The median follow-up period after liver biopsy was 2.4 years (Interquartile range; 1.0–4.9 years). HCC was detected in 19 patients during the follow-up period. The incidence rates at 1, 3 and 5 years in all patients were 1.8%, 4.3% and 9.2%, respectively, using the Kaplan-Meier method. (A) We divided these findings into two groups by the liver histology grade, F0-1 and F2-4, and the median (B) Fuc-Hpt (C) and Mac-2 bp (D) levels. These analysis were performed using the log-rank test.

The annual incidence of HCC increased with the degree of liver fibrosis, from 0.5% per year in stage F0-1 to 7.9% in stage F4. [19] We examined the correlation between the serum Fuc-Hpt or Mac-2 bp levels and the development of HCC. The median of follow-up period after liver biopsy was 2.4 years (Interquartile range; 1.0–4.9 years). HCC was detected in 19 patients during the follow-up period. The serum levels of Fuc-Hpt and Mac2-bp at the outset in patients who developed HCC were significantly higher than those in patients who did not (S1 Table). Fig 3 shows the cumulative HCC incidence rate in our cohort. The incidence rates at 1, 3 and 5 years in all patients were 1.8%, 4.3% and 9.2%, respectively, using the Kaplan-Meier method (Fig 3A). We divided these findings into two group by the liver histology grade, F0-1 and F2-4, and the median Fuc-Hpt and Mac-2 bp levels. The HCC incidence rate of the F2-4 group was significantly higher than that of the F0-1 group (p < 0.05) (Fig 3B). The HCC incidence rate of the high Mac-2 bp group was significantly higher than that of the low Mac-2 bp group (p < 0.05) (Fig 3C). Similarly, the HCC incidence rate of the high Fuc-Hpt group was significantly higher than that of the low Fuc-Hpt group (p < 0.001) (Fig 3D). When we compared Fuc-Hpt and Mac-2 bp by cox proportional hazard model, Fuc-Hpt but not Mac-2 bp was significant (S1 Table).

## Discussion

We examined the usefulness of the Fuc-Hpt and Mac-2 bp levels in evaluating liver fibrosis in patients with chronic hepatitis C. The serum levels of Fuc-Hpt and Mac-2 bp were significantly increased with the progression of liver fibrosis. The AUROC analysis revealed that the AUROCs of both Fuc-Hpt and Mac-2 bp were sufficient to evaluate the fibrotic grade in patients with chronic hepatitis C and although neither the Fuc-Hpt nor Mac-2 bp levels exceeded the values of the known fibrotic markers, the usefulness of these markers was comparable with the known fibrotic markers, such as the FIB4 index or APRI. Although Mac-2 bp is highly correlated with liver fibrosis in NAFLD patients [15], Mac-2 bp expression could be induced by viral infection or liver fibrosis. Previous reports suggest that Mac-2 bp is associated with cancer and immunoreactions [20, 21]. In the case of HCV infection, chronic inflammation occurred at Glisson’s capsule, with infiltrated lymphocytes in the liver. HCV-infected hepatocytes express HCV-derived proteins and immune cells, such as cytotoxic T lymphocytes (CTL), recognized the HCV-related antigens. The CTLs lysed the HCV-infected hepatocytes repeatedly, resulting in chronic liver damage and the development of liver fibrosis [22, 23]. Mac-2 bp expression and liver fibrosis seem to be induced during the progression of hepatic inflammation through these immunoreactions. Serum Mac-2 bp levels increased along the progression of liver fibrosis stage. However, the significant difference were not observed between F2 stage and F3 stage (Fig 1B), which might be related to the lower AUROC value of Mac-2 bp in F ≥ 3 group compared with that in F ≥ 2 group.

Because the correlation coefficients between the Fuc-Hpt or Mac-2 bp levels and the FIB4 index and APRI were weak, it encouraged us to examine the usefulness of the combination of the Fuc-Hpt or Mac-2 bp levels with the known fibrotic markers, such as the FIB4 index or APRI, in evaluating liver fibrosis. The AUROCs of the combination of the Fuc-Hpt or Mac-2 bp levels with the FIB4 index was increased compared with those of each single marker, suggesting that the combination might be a new marker for evaluating liver fibrosis. Sterling RK *et al*. reported the usefulness of the FIB-4 index [9]. They considered each odds ratio using a multiple logistic regression analysis of many factors associated with liver fibrosis, and they chose four factors that were strong and easy to use: age, AST, ALT and platelet count. Finally, they generated a simple index constructed of these factors. Consistent with a previous report, a combination of 5 to 10 basic serum biomarkers could be useful for diagnosing liver fibrosis [10]. A combination of these fibrosis marker with the Fuc-Hpt or Mac-2 bp levels -might be a novel non-invasive liver fibrosis marker.

Mac-2 bp has been used as a serum biomarker for several types of cancers, including lung and prostate carcinoma [24, 25]. Mac-2 bp has also been reported to predict the response of patients with chronic hepatitis C to peginterferon-ribavirin therapy [26]. The Mac-2 bp is an oligomer of large ring structures covered with N-glycans. It has seven potential N-glycosylation sites [20, 21, 27]. Recently, *Wisteria floribunda* agglutinin (WFA)-positive human Mac-2 bp (WFA^+^-Mac-2 bp) has attracted attention for its ability to predict liver fibrosis and the development of hepatocellular carcinoma [28–30]. WFA is a type of lectin that recognizes carbohydrate chains, including N-acetylgalactosamine. However, the authors did not determine the WFA^+^-Mac-2 bp and total Mac-2 bp levels. In this study, we estimated the Mac-2 bp levels by sandwich ELISA using a specific antibody, which is different from evaluating the serum WFA^+^-Mac-2 bp system. Although the relationship between the Mac-2 bp and WFA^+^-Mac-2 bp levels is still unknown, both could be useful for diagnosing liver fibrosis.

We demonstrated that several factors, including the Fuc-Hpt or Mac-2 bp levels, were associated with the progression of liver fibrosis in patients with chronic hepatitis C. In a previous report, there were many risk factors associated with advanced liver fibrosis in chronic hepatitis C infection, including male sex; the duration of infection; the acquisition of infection at younger age; alcohol consumption; long term immunosuppression, such as HIV and organ transplantation; HBV coinfection; insulin resistance; obesity; steatosis; high AFP levels; high AST levels; and high ALT levels [31, 32]. Interestingly, our analysis suggested that Mac-2 bp might be associated with early liver fibrosis stages. In contrast, Fuc-Hpt might be associated with advanced liver fibrosis stages. Mac-2 bp, a ligand of galectin-3, is believed to have a role in the cellular immune response. In the early stages of chronic hepatitis C, inflammation occurs in the liver and the resulting immune responses might result in an increase in Mac-2 bp secretion from the hepatocytes, leading cell-cell adhesion and fibrosis progression [33]. In contrast, fucosylation regulates the secretion of hepatic glycoproteins into the bile duct in normal polarity hepatocytes [34]. When cell polarity collapses, the cell cannot maintain its normal function because the intracellular polarized delivery is damaged. The elevated serum Fuc-Hpt levels are associated with the collapse of the hepatocyte polarity [34]. The collapse of the hepatocyte polarity might occur in late stages of chronic hepatitis C. Therefore, the increased serum Fuc-Hpt levels might reflect advanced liver fibrosis stages. Most of serum samples were collected at the point of liver biopsy before starting interferon therapy. In general, the liver cirrhosis patients were not appropriate for interferon therapy. So the number of F4 patients was small in this study, and this might weaken conclusions related to cirrhosis.

HCV infection is the major cause of HCC development worldwide [1]. In HCV infection, the risk factors for HCC development had been reported to include liver fibrosis stage, age, achieving SVR, and the serum AFP levels [35, 36]. The FIB4 index was also believed to be useful for the surveillance of HCC development in high risk patients [37]. We demonstrated that patients with elevated serum levels of Fuc-Hpt or Mac-2 bp had a higher incidence of HCC. Consistent with the FIB4 index, both Fuc-Hpt and Mac-2 bp are associated with liver fibrosis, which suggested that they have a close association with HCC development. Fuc-Hpt had been reported to be related with HCC development [38]. In addition to the fibrosis markers, both Fuc-Hpt and Mac-2 bp might be markers for the development of HCC. In this study, we observed the small number of HCC diagnoses during observation period. This is the limitation of this study to evaluate the ability of both Fuc-Hpt and Mac-2 bp for predicting for HCC occurrence.

## Conclusions

We demonstrated that both Fuc-Hpt and Mac-2 bp have potential diagnostic value for evaluating the degree of liver fibrosis in patients with chronic hepatitis C. In addition, these markers might predict the development of HCC.

## Supporting Information

S1 Table The serum Fuc-Hpt and Mac-2 bp levels at the outset in the patients with and without developing HCC. (DOCX)

S2 Table The comparison of Fuc-Hpt and Mac-2 bp as HCC predictive marker. (DOCX)
