# Supplementary material for: Evaluation of Fucosylated Haptoglobin and Mac-2 Binding Protein as Serum Biomarkers to Estimate Liver Fibrosis in Patients with Chronic Hepatitis C
Source: PLoS One. 2016 Mar 22;11(3):e0151828. doi: 10.1371/journal.pone.0151828 (PMC4803196; doi:10.1371/journal.pone.0151828)
Supplement: S1 Table — (DOCX) [file pone.0151828.s001.docx]

| Factor | Median [Interquartile range] | | P value |
| --- | --- | --- | --- |
|  | No development of HCC  (n = 298) | Development of HCC  (n = 19) |  |
| Fuc-Hpt, U/mL | 517 [298 – 1019] | 1163 [726 – 2421] | 0.0109* |
| Mac-2 bp, ng/mL | 1705 [1012 – 2987] | 3241 [2062 – 4071] | 0.0020* |
