# Supplementary material for: Evaluation of Fucosylated Haptoglobin and Mac-2 Binding Protein as Serum Biomarkers to Estimate Liver Fibrosis in Patients with Chronic Hepatitis C
Source: PLoS One. 2016 Mar 22;11(3):e0151828. doi: 10.1371/journal.pone.0151828 (PMC4803196; doi:10.1371/journal.pone.0151828)
Supplement: S2 Table — (DOCX) [file pone.0151828.s002.docx]

| Factor | Category | Univariate analysis | | | Multivariate analysis | | |
| --- | --- | --- | --- | --- | --- | --- | --- |
|  |  | HR | 95% CI | P value | HR | 95% CI | P value |
| Fuc-Hpt, U/mL | ≤ 559 (median) vs. > 559 | 8.7 | 2.5 - 56 | < 0.05* | 7.2 | 1.8 – 48 | < 0.05* |
| Mac-2 bp, ng/mL | ≤ 1847 (median) vs. > 1847 | 3.2 | 1.1 - 11 | < 0.05* | 1.6 | 0.54 – 5.8 | 0.43 |

HR; hazard ratio
